# Comparison of CPAP and HFNC in Management of Bronchiolitis in Infants and Young Children

**DOI:** 10.3390/children4040028

**Published:** 2017-04-20

**Authors:** Majken Bisgaard Pedersen, Signe Vahlkvist

**Affiliations:** Pediatric Department, Hospital of South West Jutland, Finsensgade 35, 6700 Esbjerg, Denmark; Majkenpedersen@gmail.com

**Keywords:** bronchiolitis, non-invasive ventilation, continuous positive airway pressure (CPAP), high flow nasal cannula (HFNC), children

## Abstract

Continuous positive airway pressure (CPAP) has been used in infants with bronchiolitis for decades. Recently, high flow nasal cannula (HFNC) therapy was introduced We conducted a retrospective study of treatment with CPAP vs. HFNC between 2013 and 2015, comparing the development in respiratory rate, fraction of inspired oxygen (FiO2) and heart rate, treatment failure, duration of treatment, and length of hospital stay. A sample size of 49 children were included. Median age was 1.9 months. Median baseline pCO_2_ was 7.4 kPa in both groups, respiratory rate per minute was 57 vs. 58 (CPAP vs. HFNC). Respiratory rate decreased faster in the CPAP group (*p* < 0.05). FiO2 decreased in the CPAP group and increased in the HFNC group during the first 12 h, whereafter it decreased in both groups. (*p* < 0.01). Heart rate development was similar in both groups. Twelve children (55%) changed systems from HFNC to CPAP due to disease progression. There was no difference in length of treatment, hospital stay, or transmission to intensive care unit between the groups. CPAP was more effective than HFNC in decreasing respiratory rate (RR) and FiO2. No differences were observed in length of treatment or complications. Further studies should be conducted to compare the efficacy of the two treatments of bronchiolitis, preferably through prospective randomized trials.

## 1. Introduction

Every winter, bronchiolitis due to respiratory syncytial virus (RSV) or other viruses is a common cause of hospitalization in infants and young children. In Denmark, approximately 3% of children under 2 years of age have been hospitalized due to RSV infection [1]. Clinically, bronchiolitis presents with coughing, wheezing, retractions, mucus blocking of the airways, increased respiratory rate and work, need of oxygen supply, and feeding difficulties. In some cases the condition proceeds to respiratory failure and need for ventilatory support.

In Denmark, treatment with continuous positive airway pressure (CPAP) is standard for infants with moderate-severe bronchiolitis and respiratory fatigue. The mechanism of CPAP is not fully understood, but the positive pressure is thought to decrease the inspiratory resistance and thereby relieve muscuar work and improve alveolar ventilation [2] It has been shown to abolish airway occlusion and increase diaphragmatic tone [3] CPAP has been shown to be superior to standard care in improving clinical outcomes and decreasing pCO_2_ [4,5]. However, evidence is scarce and two reviews, among them a Cochrane review [6,7], have concluded that more randomized trials are needed in evaluating the efficacy of CPAP in bronchiolitis.

In the past decade, high flow nasal cannula (HFNC) therapy has been introduced as an alternative method for the management of respiratory distress due to bronchiolitis. HFNC delivers a heated, humidified airflow, and the FiO2 can be varied between 21% and 100% [8].

HFNC, like CPAP, is a high flow system and is able to generate a positive end expiratory pressure, but unlike CPAP it does not have a valve [9]. HFNC is suggested to reduce the upper airway dead space and resistance [10,11]. HFNC is considered a less invasive treatment than CPAP, better tolerated by the patients, and easier to handle by the staff [12]. In some studies, HFNC has been shown to be more efficient than standard care (e.g., CPAP) [12,13,14]. However, more evidence is needed to show the efficacy of HFNC [15,16].

In the season of 2014–2015, HFNC was gradually introduced as an alternative method to CPAP at the Pediatric Department of the Hospital of South West Jutland, Denmark. Prior to that, CPAP was the primary choice for noninvasive respiratory support. We conducted a study of CPAP vs. HFNC in the management of respiratory care in infants and young children with bronchiolitis, using data from the seasons 2013–2014 and 2014–2015.

## 2. Materials and Methods

### 2.1. Settings and Patients

The Pediatric Department at the Hospital of South West Jutland is a broad pediatric department which serves children and adolescents aged 0–17 years with pediatric and surgical conditions. Semi-intensive care with CPAP/HFNC can be managed, and intubation/mechanical ventilation can be initiated by the local intensive care unit, prior to transferal to the nearest Pediatric Intensive Care Unit (PICU).

For the present study, bronchiolitis was defined as acute respiratory illness in children <2 years of age, with characteristic patterns of wheeze, cough, and respiratory distress. Criteria for respiratory support were, according to local guidelines, a clinical decision based on signs of respiratory failure: high respiratory rate (RR), retractions, recurrent apnea, hypercapnia and acidosis.

Failure of treatment was assessed by a physician when the clinical response to the given treatment was not sufficient. In case of failure, therapy could be changed to CPAP or mechanical ventilation, and the child was eventually transferred to PICU. During CPAP/HFNC treatment, all children were intensively observed by a trained nurse with heart rate (HR), RR, FiO2 and saturation scheduled hourly. Blood tests including capillary gas values (pH, pCO_2_) were measured prior to treatment, and controlled as needed.

Inclusion criteria were: children aged 0–2 years, with bronchiolitis, and need for non-invasive respiratory support. The study period was from October 2013 to May 2015.

Exclusion criteria were: need of invasive respiratory support, monitoring in an intensive care unit at time of admission, or a history of chronic respiratory disease.

The electronic patient journal system was searched for patients registries with ICD10 diagnose codes DJ205, DJ210, DJ211, DJ219, DJ121 or procedure code BGFC32 in the winters of 2013–2014 and 2014–2015 to identify children fulfilling the inclusion criteria.

For CPAP, we used a binasal prong with a Benveniste valve (Dameca^®^, Copenhagen, Denmark) connected to a humidifier (Fisher & Paykel Healthcare^®^, Auckland, New Zealand). According to our guidelines, the initial flow was 14 L/min.

For HFNC, we used an Optiflow Junior (Fisher & Paykel Healthcare^®^, Auckland, New Zealand) to deliver a heated, humidified flow. We used three sizes of nasal prongs, according to the weight of the child. According to the guidelines from the manufacturer, the initial flow was 8 or 12 L/min, corresponding the weight of the child.

Oxygen supply was delivered as needed to both systems to maintain a SpO_2_ above 92%.

Chest X-ray and nasopharyngeal mucus polymerase chain reaction (PCR) analysis for RSV and metapneumovirus were routinely conducted in patients in need of respiratory support.

### 2.2. Data Management

From patient journals, data regarding age, sex, prematurity, weight, RSV status, duration of symptoms, device, blood gas analysis, treatment failure, duration of treatment, and length of hospital stay were registered.

For this study, RR, FiO2 and HR were noted at treatment start time, and after 6, 12, 18, 24 and 48 h. In case of a shift of device, the new device was noted from time of shift. In case of admission to PICU (with or without invasive respiratory support) the case was noted as a failure, and data collection was withdrawn from the time of failure.

### 2.3. Statistical Analysis

For group comparisons, Wilcoxon rank-sum test was used because data were generally not normally distributed. Chi-squared test was used for binary outcomes.

Development in HR, RR and FiO2 was measured using a variance-components model with maximum likelihood estimation. Device, time, age, and sex, as well as interaction between time and device were chosen as explanatory variables. A squared link of time was also included to investigate nonlinear effects. The model was fitted with significant variables using simple backward elimination. Post-estimation analysis was conducted using residual diagnostics. For all analyses, significance level was defined as *p* < 0.05.

All statistical analyses were performed using STATA version 10 statistical software (StataCorp, College Station, TX, USA).

### 2.4. Ethical Approval

All procedures performed in studies involving human participants were in accordance with the ethical standards of the institutional and national research committee and with the 1964 Helsinki Declaration and its later amendments or comparable ethical standards. For this type of study, formal consent is not required. Permission to gather patient journal information was given by the Danish Health Authority (3-3013-1056/I/reference SMFS) according to the current legislation.

## 3. Results

Fourty-nine cases were reviewed and met the inclusion criteria. No cases were identified between May 2014 and October 2014. During the two winter seasons between 2013 and 2015, 27 children were treated with CPAP and 22 with HFNC. In the first period, all children were in CPAP; in the second period 22 and 9 patients were treated by HFNC and CPAP, respectively. No differences were observed in age, weight, gender, gestational age, or RSV status between the two groups (Table 1). Symptom duration was reported one day longer at admission for the HFNC group, which was peri-significant. Both groups were similar for X-ray description (±atelectasis), RR, HR, FiO2, and pCO_2_ at baseline (Table 1).

During the second season, where both systems were available, 31 children were included, of which 22 were treated with HFNC and 9 with CPAP. The CPAP children had a median age of 1.3 (0.6–1.9) months vs. 2.2 (1.5–5.5) months in HFNC group, although the difference did not reach significance (*p* = 0.07). There was no difference in the median baseline RR (56 (48–66) vs. 58 (50–60)) or pCO_2_ (7.2 (6.9–8.0) mmHg vs. 7.4 (5.6–8.0) mmHg) between the two groups included in the second season.

No differences were observed in treatment length, hospitalization duration or transferal to PICU (Table 2). More than half of the children from the HFNC group were changed to CPAP therapy (*p* < 0.001). For patients experiencing system failure, median time from treatment start to change in system was 18 h, whereas median time to referral to PICU were 31 h, with no difference between the two groups. The four HFNC children which were transferred to PICU had all been changed to CPAP prior to transferal, with a median time of 11.5 (5–25) h from change of system to transferal.

**Figure 1:**
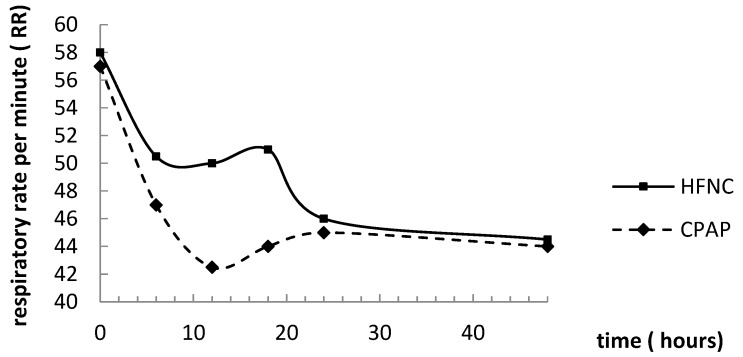

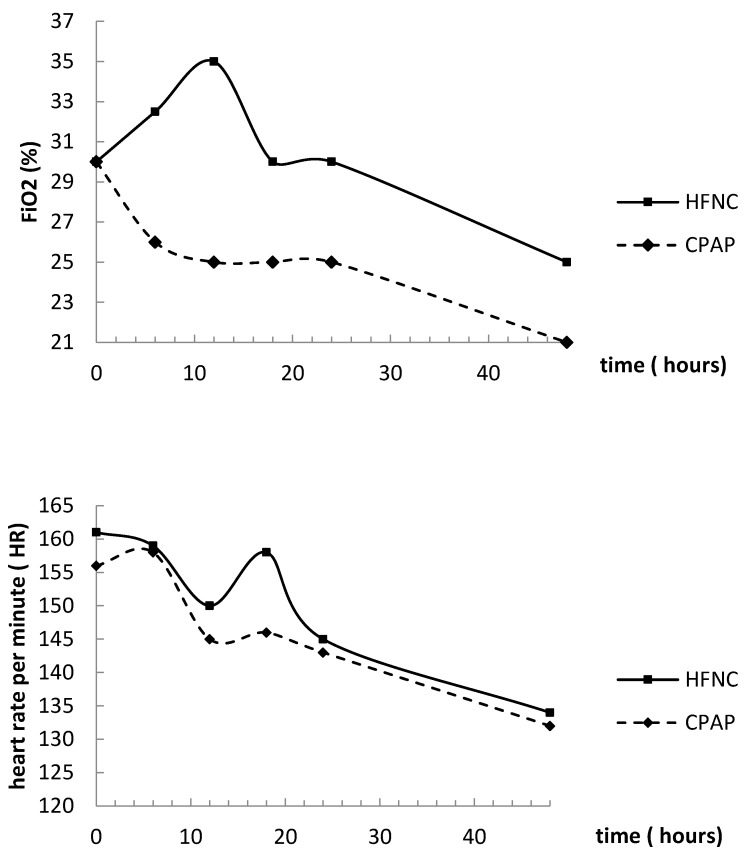
Median respiratory rate (RR), FiO2 and heart rate (HR) during 48 h of treatment. RR and FiO2 decreased significantly more in the CPAP group. HR decreased equally in both groups.

Using the variance-component model, we found that RR decreased over time in both groups. It was found that the RR declined faster in the CPAP group (0 < 0.05), independently of age and gender (Figure 1).

FiO2 declined over time in the CPAP group. In the HFNC group, FiO2 increased during the first 12 h and decreased for the rest of the treatment (Figure 1). The difference in FiO2 development between the groups was highly significant (*p* < 0.01). Age had a significant effect on FiO2, and was thus kept in the model.

HR declined significantly with time in both groups (Figure 1). There was no difference between systems, and no effect of any other explanatory variable.

## 4. Discussion

Our data suggest that CPAP was superior to HFNC with respect to reducing RR and need of oxygen supply in infants with bronchiolitis. This is in opposition to Metge et al., who found no difference with respect to RR development [17]. On the contrary, Metge et al. found, in concordance with our findings, a higher FiO2 in the HFNC group, although it did not reach significance. The differences may be explained by differences in design and management of outcomes, as well as in patient population—the children in our study being a few weeks older. In accordance with our findings, Milesi et al. recently found a higher failure rate of HFNC compared to CPAP in treatment of infants with bronchiolitis [18].

In our study, more than half of the patients that started treatment with HFNC were changed to CPAP treatment, suggesting a high level of treatment failure with HFNC. It should be remembered that despite the diagnosis of system failure based on systematic observations of RR, respiratory work and hypercapnia, there might have been a bias in the clinical decision towards favoring CPAP which is the well-known treatment. On the contrary, as shown in Figure 1, both RR and FiO2 improved after approximately 24 h, where many children were converted to CPAP treatment.

The optimal pressure level of CPAP is 7 cm H_2_O. HFNC has been shown to be able to generate a pressure of 4–5 cm H_2_O^9^. The actual positive end expiratory pressure (PEEP) of both systems is, however, influenced by many factors e.g., whether the child is able to breath with a closed mouth and to cooperate with the system. In HFNC, the available pressure also depends on the size and placement of the nostril prongs, whereas CPAP, when placed correctly, is a hermetical system. We suggest that our findings may be explained by CPAP’s better ability to keep an optimal and constant PEEP.

As mentioned earlier, in Denmark, CPAP is first line treatment for moderate-severe bronchiolitis, in opposition to conservative treatment (oxygen, hydration etc.). However, even though a few studies have shown that CPAP is superior to the conservative treatment, evidence is scarce. Only one randomized study exists, a cross-over study comparing CPAP with standard care^4^. Our findings of CPAP being superior to HFNC, with respect to lowering RR and FiO2, may contribute to the evidence of CPAP in the management of bronchiolitis.

Our study is limited by its retrospective, unrandomized design. However, there was no difference in age, gender, and disease severity, measured by pCO_2_, RR, pulse, and FiO2, at the inclusion time. One might speculate that, during the second season, HFNC was chosen for the milder cases and CPAP for the severe cases, but this was not the case, although there was a nonsignificant tendency of choosing CPAP for the youngest children. We therefore conclude that the two groups were comparable with respect to patient characteristics and disease severity. Further limitations of this study are the lack of standardized measurements that could be planned in a prospective design (e.g., a standardized clinical score and control pCO_2_). The limitations in this study emphasize the need of prospective randomized trials comparing the effect of the two systems in treating bronchiolitis.

Even though our data showed the better effects of CPAP, HFNC may be a good alternative for children with less severe bronchiolitis. Our study found no differences in treatment length, hospitalization length, or referral to PICU, and indicates that HFNC is safe, if carefully monitored. Future studies may be able to focus on subgroups of patients benefitting from the two systems.

## 5. Conclusions

CPAP was superior to HFNC in lowering RR and FiO2 in infants with bronchiolitis. More than half of the children treated with HFNC were changed to CPAP treatment due to suspected treatment failure. There were no differences between HFNC and CPAP in treatment length, hospitalization length or transferal to PICU.

## Figures and Tables

**Table 1:** Descriptive statistics.

	nCPAP	HFNC	*p*-value
*n*	27	22	
Gender, boys *n* (%)	13 (48)	8 (36)	ns
Age, months, median (IQ range)	1.7 (1.2–3.0)	2.2 (1.53–5.33)	ns
Gestational age at birth, weeks + days, median (IQ range)	39 + 3 (38 + 2–40 + 0)	40 (37 + 3–40 + 3)	ns
Weight, kg, median (IQ range)	5.2 (3.9–6.6)	5.3 (4.7–6.7)	ns
Symptom duration, days , median (IQ range)	4 (2–5)	5 (4–7)	0.052
RSV positive, *n* (%)	24 (89.9)	19 (90.5)	ns
Atelectasis, *n* (%)	7 (30.4)	6 (33.3)	ns
pCO_2_, kPa, median (IQ range)	7.4 (5.6–8.3)	7.4 (6–8.3)	ns
Respiratory Rate per minute, median (IQ range)	57 (48–62)	58 (50–60)	ns
FiO2, % (IQ range)	30 (21–35)	30 (25–35)	ns
Pulse, per minute	156 (148–170)	161 (148–178)	ns

IQ: Interquartile; nCPAP: Nasal continuous positive airway pressure; HFNC: High flow nasal cannula; RSV: Respiratory syncytial virus; FiO2: fraction of inspired oxygen. Ns: non-significant.

**Table 2:** Duration of treatment, hospital stay and system failure.

	nCPAP	HFNC	*p*-value
Duration of treatment, median (hours)	93.5 (58.3–163.0)	93.0 (70–146)	ns
Duration of hospitalization, median (days)	7 (4–10)	8 (5–11)	ns
Device failure (shift to opposite), *n* (%)	0 (0%)	12 (55%)	<0.001
Device failure (referral to PICU), *n* (%)	4 (15%)	5 (24%)	ns

PICU: Pediatric Intensive Care Unit.
